# Digestion of Yeasts and Beta-1,3-Glucanases in Mosquito Larvae: Physiological and Biochemical Considerations

**DOI:** 10.1371/journal.pone.0151403

**Published:** 2016-03-23

**Authors:** Raquel Santos Souza, Hector Manuel Diaz-Albiter, Vivian Maureen Dillon, Rod J. Dillon, Fernando Ariel Genta

**Affiliations:** 1 Laboratory of Insect Biochemistry and Physiology, Oswaldo Cruz Institute, FIOCRUZ, 4365 Brasil Av, Leonidas Deane Building, room 207, Manguinhos, Rio de Janeiro, Brazil, 21040–360; 2 Institute of Integrative Biology, Biosciences Building, University of Liverpool, Crown Street, Liverpool, L69 7ZB, United Kingdom; 3 Division of Biomedical and Life Sciences, Furness Building, Lancaster University, Bailrigg, Lancaster, LA1 4YG, United Kingdom; 4 National Institute of Science and Technology for Molecular Entomology, 373 Carlos Chagas Filho Av., Center for Health Science, Building D, Basement, room 5, Cidade Universitária, Rio de Janeiro, Brazil, 21941–590; New Mexico State University, UNITED STATES

## Abstract

*Aedes aegypti* larvae ingest several kinds of microorganisms. In spite of studies regarding mosquito digestion, little is known about the nutritional utilization of ingested cells by larvae. We investigated the effects of using yeasts as the sole nutrient source for *A*. *aegypti* larvae. We also assessed the role of beta-1,3-glucanases in digestion of live yeast cells. Beta-1,3-glucanases are enzymes which hydrolyze the cell wall beta-1,3-glucan polyssacharide. Larvae were fed with cat food (controls), live or autoclaved *Saccharomyces cerevisiae* cells and larval weight, time for pupation and adult emergence, larval and pupal mortality were measured. The presence of *S*. *cerevisiae* cells inside the larval gut was demonstrated by light microscopy. Beta-1,3-glucanase was measured in dissected larval samples. Viability assays were performed with live yeast cells and larval gut homogenates, with or without addition of competing beta-1,3-glucan. *A*. *aegypti* larvae fed with yeast cells were heavier at the 4^th^ instar and showed complete development with normal mortality rates. Yeast cells were efficiently ingested by larvae and quickly killed (10% death in 2h, 100% in 48h). Larvae showed beta-1,3-glucanase in head, gut and rest of body. Gut beta-1,3-glucanase was not derived from ingested yeast cells. Gut and rest of body activity was not affected by the yeast diet, but head homogenates showed a lower activity in animals fed with autoclaved *S*. *cerevisiae* cells. The enzymatic lysis of live *S*. *cerevisiae* cells was demonstrated using gut homogenates, and this activity was abolished when excess beta-1,3-glucan was added to assays. These results show that live yeast cells are efficiently ingested and hydrolyzed by *A*. *aegypti* larvae, which are able to fully-develop on a diet based exclusively on these organisms. Beta-1,3-glucanase seems to be essential for yeast lytic activity of *A*. *aegypti* larvae, which possess significant amounts of these enzyme in all parts investigated.

## Introduction

*Aedes aegypti*, among other species of the genera *Aedes*, is the main vector of several pathogenslike Dengue, Urban Yellow Fever, Chikungunya, West Nile and Zika viruses, whose endemic areas include 40% of human populations worldwide (2.5 billion people) [1, 2]. In spite of being considered diseases restricted to tropical countries, recent global warming has increased concerns about their spread to regions with temperate climate [3], including reports of West Nile virus in Europe, Asia, North America and Australia [4].

Current main strategies for fighting these diseases rely on vector control, as there are no vaccines commercially available. Historically, the control of mosquitoes has been done with chemical insecticides, which are losing their potential effectiveness due to appearance of resistant populations [5]. New strategies for control of vector populations as transgenic mosquitoes, transfection of insects with *Wolbachia* or paratransgenesis have been proposed and are currently under evaluation [6–8]. Interestingly, some of these strategies depend on rearing massive amounts of insects and, therefore, mosquito nutrition has become a strategic point of investigation.

The haematophagic bevaviour of adult female *A*. *aegypti* and the fact that the initial site for development and transmission of pathogens by this insect is the intestine, had led to several studies of its digestive physiology [9–12]. Comprehensibly, those studies have focused the physiology of female adults, and larval digestion is known to a lesser extent [9, 13–14].

Interestingly, burden of *A*. *aegypti*-transmitted diseases is primarily determined by the occurrence of larval breeding sites [15]. Thus, knowledge of larval physiology and biochemistry can result in new insights for vector control. *A*. *aegypti* larvae are considered as detritivores, ingesting solid particles from liquid media and scraping solid material from surfaces. Among the particles ingested by mosquito larvae several microorganisms, such as bacteria, fungi, protozoa and rotifers have been found [16–21], but the mechanisms used by larvae for breakdown of these nutritional sources remain largely unknown.

Recent understanding of the importance of gut microbiota in several aspects of insect physiology [22] resulted in more detailed investigations of the role of bacteria in development and vectorial capacity of mosquitoes. For example, it was demonstrated the dependence of *Aedes aegypti*, *Anopheles gambiae* and *Georgecraigius atropalus* larvae on gut bacteria for full development [23]. In spite of that, the exact mechanisms of interaction between these organisms was not fully investigated, as beneficial effects of ingested bacteria might be of nutritional, immunological or even endocrinological nature. In this respect, a deep understanding of interactions between specific microorganisms and mosquito larvae is still lacking.

The main objective of this work was to investigate physiological consequences of yeast ingestion by *A*. *aegypti* larvae, using *Saccharomyces cerevisiae* as model nutrient source. Yeasts are a more defined food source, antibiotic free and less likely to transmit pathogens to the insects than the standard cat or animal food which is used to raise larvae in regular mosquito colonies [24–26]. We discovered that *A*. *aegypti* larvae could nourish exclusively from live *S*. *cerevisiae* cells, revealing that this insect bears mechanisms for yeast cell wall breakdown and full acquisition of nutrients from this microorganism. Accordingly, we showed *in vitro* that larval gut homogenates have lytic activity against live *S*. *cerevisiae* cells.

Beta-1,3-glucanases hydrolyse glicosidic bonds in beta-1,3-glucans, which are the major polysaccharide component of the yeast cell wall. We investigated the effects of a *S*. *cerevisiae* exclusive diet on larval beta-1,3-glucanase activity, and competition experiments revealed that this enzyme is crucial for the larval lytic activity against this microorganism. These findings, besides unravelling new basic physiological aspects of culicid larvae, could help in the establishment of better defined, pathogen free artificial diets for large-scale mosquito larvae rearing in future.

## Materials and Methods

### Insects rearing and maintenance

*Aedes aegypti* eggs from the Rockfeller strain were obtained from the colony of the Laboratory of Physiology and Control or Arthropod Vectors (LAFICAVE/IOC-FIOCRUZ; Dr Denise Valle and Dr José Bento Pereira Lima). Insects were reared until adult stage in the Laboratory of Insect Biochemistry and Physiology (LABFISI, IOC/FIOCRUZ) at 27±2°C and 70±10% relative humidity with a 12-h light/12-h dark cycle. To obtain synchronized developing larvae, hatching was induced by adding 100 mL of distilled water into 200 mL plastic cups containing eggs and then incubating at 28°C for 30 minutes. After incubation, first instar larvae (n = 80) were transferred together to plastic bowls containing 100 mL of dechlorinated water and 0.1 g of cat food (Whiskas®, Purina, Brazil) and kept at 26±1°C until adult stage. The food was added only once in the beginning of each experiment. Larvae which received cat food are considered the control group.

*Saccharomyces cerevisiae* S14 was kindly donated by Professor Pedro Soares de Araújo (Chemistry Institute, University of São Paulo, Brazil). For feeding experiments with live *S*. *cerevisiae*, a single colony was transferred into 3–5 mL of liquid Sabouraud medium [27] and incubated overnight at 30°C under shaking at 100 rpm. After overnight incubation, 100 uL of culture were subpassaged into 50 mL of Sabouraud medium and incubated overnight at 30°C under shaking at 100 rpm. 50 mL of cultures were then centrifuged (7,500 x *g*, 30 min, 4°C) and the supernatant was discarded. All cells were then suspended in water and released into larval cups. A similar experiment was performed autoclaving the cells (120°C, 20 min, 1.5 atm) before larval feeding.

### Biological parameters

Initially, we investigated if a yeast diet could have an impact in development of fourth instar larvae of *Aedes aegypti*. With this objective, recently molted 4^th^ instar larvae were fed on live *Saccharomyces cerevisiae* cells until the prepupae phase. To investigate if *A*. *aegypti* could fully-develop when feeding exclusively on cells of this yeast species, recently hatched first instar larvae were transferred to a bowl containing yeasts as the sole food source. Larval and pupal mortality, pupation and emergence were monitored and recorded daily. Fourth instar larvae, pupae, and male and female adults were weighed individually or in pools of 10 individuals each. Pupation and emergence data were plotted and compared by the Log-rank (Mantel-Cox) Test. Mortality and weights were expressed as means ± SEM and non-transformed data were compared by ANOVA or pairwise t-tests.

### Preparation of samples for enzymatic assays

Larvae were immobilized by placing them on ice, after which they were dissected in cold 0.9% (w/v) NaCl. Parts dissected in each larva were the head, gut and rest of body. Heads and rest of bodies were homogenized in MilliQ water with aid of a micro tube pestle (Model Z 35, 997–1, Sigma, USA), using a ratio of 100 μL of water per 10 insects. Guts were homogenized in cold MilliQ water containing 20 mM phenylmethylsulfonyl fluoride (PMSF), 20 μM Pepstatin A and 20 μM trans-epoxysuccinyl-L-leucylamido(4-guanidino)butane (E-64). All samples were centrifuged for 10 min at 14,000 x *g* at 4°C. Both pellets and soluble fractions were stored at -20°C until used as enzyme source for enzymatic assays.

### Yeast viability assays

To test if larval gut contents have some influence in live yeast cells, we performedassays incubating these two materials mixed and followed yeast viability. Gut soluble fraction was prepared as above and filtered through a 0.45 μm PVDF syringe filter (Millipore Code. JBR6 103 14 Lot. B2MN40511) and then incubated at 30°C with 10 colony-forming units (CFUs)/μL of live *S*. *cerevisiae* cells in 10 mM citrate-sodium buffer pH 7.0.

After different time points, assays were sampled and aliquots were plated onto solid Sabouraud medium (1% w/v yeast extract, 1% w/v peptone, 1% w/v dextrose, 2% w/v Agar). After overnight incubation at 30°C, colonies were counted. Cell stability under assay conditions was confirmed by using controls without enzyme.

### Enzymatic assays and protein quantitation

β-1,3-glucanase activity in *Aedes aegypti* larvae was determined by measuring the release of reducing groups from 0.25% (w/v) laminarin from *Laminaria digitata*, (SIGMA Cat. no. L9634) in a thermocycler with a modified bicinchoninic acid reagent according to ref. [28]. All assays were performed at 30°C under conditions such that activity was proportional to protein concentration and time. Controls without enzyme or without substrate were included. One unit of enzyme (U) is defined as the amount that hydrolyses 1 μmol of substrate (or bonds)/min. Protein concentration was determined according to [29] using ovalbumin as a standard.

To test if feeding with yeasts could change beta-1,3-glucanase expression, we compared activities in all parts of *A*. *aegypti* 4^th^ instar larvae reared on live or autoclaved *S*. *cerevisiae* cells with levels found in larvae fed on cat food. Comparisons between means of two independent groups were done with a pairwise t test. Results are expressed as the group mean ± SEM.

### Yeast cell counts

To confirm that *A*. *aegypti* larvae are actively ingesting live *S*. *cerevisiae* cells, and not merely filtrating released molecules from broken or dead cells, we decided to check the integrity and viability of the yeasts in our experimental conditions. During the preparation of the experimental diets, the yeasts, after growing on liquid Sabouraud media, are centrifuged and ressuspended in water. We decided to count viable cells using Trypan Blue staining and by light microscopy after these treatments to check viability as below.

*S*. *cerevisiae* cultures (45 mL) were prepared in Sabouraud liquid media as described previously and then centrifuged (7,500 x *g*, 40 min, 4°C). The supernatant was discarded and cells were resuspended in the same volume of Sabouraud liquid media or water. Ten microliter aliquots of each suspension were withdrawn and then combined with 90 μL of PBS. These samples were mixed with 100 μL of a 0.4% (w/v) Trypan Blue solution in PBS and then 15 μL were loaded on a Neubauer chamber (hemocytometer), where dead and live cells were counted in a light microscope (400 x magnification). In one experiment, yeast cells ressuspended in water were kept at 26°C for 24 hours before staining and counting.

For counting yeast cells ingested by *A*. *aegypti* larvae, insects were raised on cat food as described previously until they reached the fourth larval instar. Then larvae were transferred to a bowl with *S*. *cerevisiae* cells as food source as described, and after different time points larvae were withdrawn from the pots and dissected. Entire guts were homogenized in 100 μL PBS, combined with Trypan Blue and then live and dead yeast cells were counted as above.

### Food protein and sugar contents

For quantitations in *S*. *cerevisiae*, cells were grown in Sabouraud liquid media as described and 45 mL of culture were centrifuged (7,500 x *g*, 30 min, 4°C). Supernatant was discarded and cells were ressuspended in 5 mL of water. Ten microliter aliquots were withdrawn for protein and sugar measurements. For quantitations in cat food samples, 0.1 g of cat food was homogeneized in 1 mL water and 20 μL aliquots were withdrawn for measurements. Proteins were determined with the bicinchoninic acid method [30] and total sugars were measured with the phenol-sulfuric method [31]. Due to the presence of insoluble material, cat food samples submitted to reaction with BCA were centrifuged (quick spin) before absorbance readings.

### Statistical analysis

Linear regressions were performed using Microsoft Excel (Microsoft). Statistical comparisons were made using GraphPad Prism software (version 5.0, GraphPad Software Inc.). Significance was considered when *p*<0.05.

## Results

**Fig 1:**
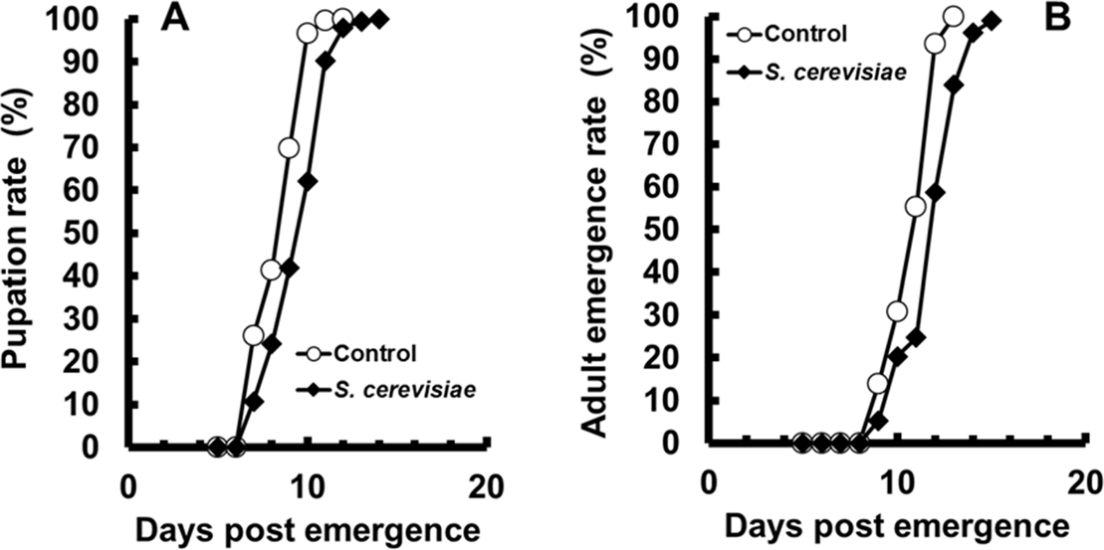
Life parameters of *Aedes aegypti* larvae fed since egg hatching exclusively with cat food or *Saccharomyces cerevisiae* live cells. Percentage of pupation (A) and percentage of emergence of adults (B). Figures are means of 4 experiments with 80 larvae each.

*A*. *aegypti* 4^th^ instar larvae fed on live *Saccharomyces cerevisiae* cells reached the end of the larval stage with significantly higher weights when compared to controls (*p* < 0.05, unpaired t-test, n = 3, Table 1). *A*. *aegypti* raised from eggs on live *S*. *cerevisiae* cells resulted in larvae heavier than controls (*p* < 0.05, unpaired t-test, n = 6, Table 1).However, pupae and female adults derived from these larvae had similar weights when compared to controls (*p* > 0.05, unpaired t-test, n = 6, Table 1). Yeast fed male adults had weights significantly higher than controls (*p* < 0.01, unpaired t-test, n = 6, Table 1). We observed a small but significant delay in both pupation and adult emergence (p < 0.05, Log-rank (Mantel-Cox) Test, n = 320, Fig 1), but no significant changes in larval or pupal mortality (*p* > 0.05, unpaired t-test, n = 6, Table 1).

**Table 1:** Biological life parameters of *Aedes aegypti* raised on different diets. Cat food—insects fed on control diet. Yeast—insects fed on live *S*. *cerevisiae* cells. Weights are presented in mg and mortalities as percentages.

Parameter	Cat Food	Yeast
Larval body weight (1)	1.7 ± 0.1	3.3 ± 0.4 *
Larval body weight (2)	3.20 ± 0.05	5.5 ± 0.1 *
Pupal body weight (2)	6.6 ± 0.4	7.4 ± 0.5
Adult female body weight (2)	2.0 ± 0.2	2.1 ± 0.2
Adult male body weight (2)	0.9 ± 0.1	1.7 ± 0.2 **
Larval mortality (2)	7 ± 2	6 ± 1
Pupal mortality (2)	12 ± 4	17 ± 5

Insects were raised in groups from eggs on cat food and exposed to different diets only during the 4 ^th^ larval instar. Figures are means ± SEM of 3 experiments with 40 larvae each.

* *p* < 0.05

Insects were raised on different diets thoughout the entire larval development. Figures are mean ± SEM of 6 experiments with 80 insects each.

* *p* < 0.05

** *p* < 0.01.

**Fig 2:**
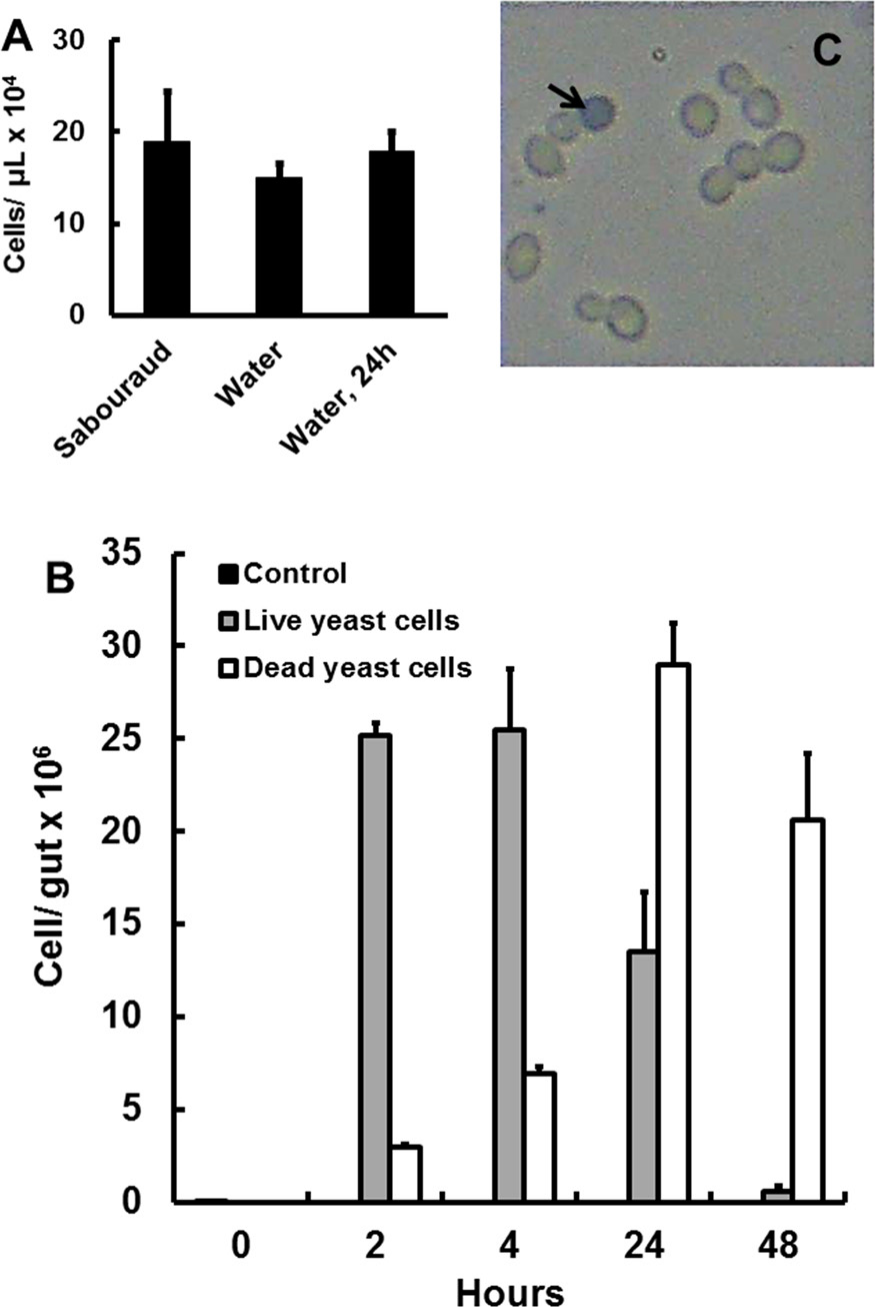
Cell counts during preparation of yeast-based diets and their ingestion by *Aedes aegypti* larvae. (A) Total counts of yeast cells after centrifugation of *Saccharomyces cerevisiae* liquid cultures and resuspension in media (Sabouraud), water and after keeping the resuspended cells in water for 24 hours. Figures are means ± SEM of 9 experiments each. (B) Time progression of yeast cells total counts in the gut of insects fed with cat food (Control), and live/dead yeast cell counts in the gut of insects fed with *S*. *cerevisiae* diet. Figures are means ± SEM of 5 samples with one larva each (C) Illustrative image of *S*. *cerevisiae* cells recovered from the gut of *A*. *aegyti* larvae fed with the yeast-based diet. The black arrow shows a dead yeast cell (Trypan Blue staining). See Material and Methods for details.

Viable cell counts revealed that ressuspension in water does not affect the number or viability of yeast cells (p > 0.05, unpaired t-test, n = 9, Fig 2A). Yeast cells remain viable even after being incubated in water for 24 hours (p > 0.05, unpaired t-test, n = 9, Fig 2A), which suggests that larvae have been exposed to live cells throughout our experiments.

Counting of yeast cells inside the gut of 4^th^ instar larvae which were exposed to *S*. *cerevisiae* diets revealed that the insects have ingested a significant amount of cells already at the first time point analysed (2 hours; Fig 2B). During 48 hours of exposure of larvae to the yeast diet, the total number of ingested cells does not dramatically change. However, a significant decrease in viable cells occured after 24 hours, with an increase of dead cells (Fig 2B and 2C). At the same conditions, control insects maintained on cat food showed no yeasts inside the gut (Fig 2B). Taken together, these results clearly show that, in spite of some changes in development, *A*. *aegypti* can nourish and fully-develop from alive *S*. *cerevisiae* cells.

To have a better understanding of possible reasons for the observed changes in development when *A*. *aegypti* larvae are raised in live yeast cells, we compared the protein and sugar amounts in the yeast diet to the amounts present in the regular cat food which was given to controls. The yeast diet contains respectively 11.6 and 3.2 times more protein and sugar than the cat food, when we compare the amounts which were given to each group (Table 2).

**Table 2:** Nutritional parameters of the different diets tested for *Aedes aegypti* larvae. Cat food was used to raise insects in control conditions. Yeast cells (*Saccharomyces cerevisiae*) were grown in liquid Sabourad media and offered to larvae as described. Figures correspond to protein and sugar quantities which are present in the amounts of food used to raise A. aegypti adults from eggs. See Material and Methods for details.

Nutrients	Cat Food	Yeast
Protein (mg)	6.9 ± 0.8	81 ± 4
Total sugars (mg)	54 ± 10	170 ± 20

**Fig 3:**
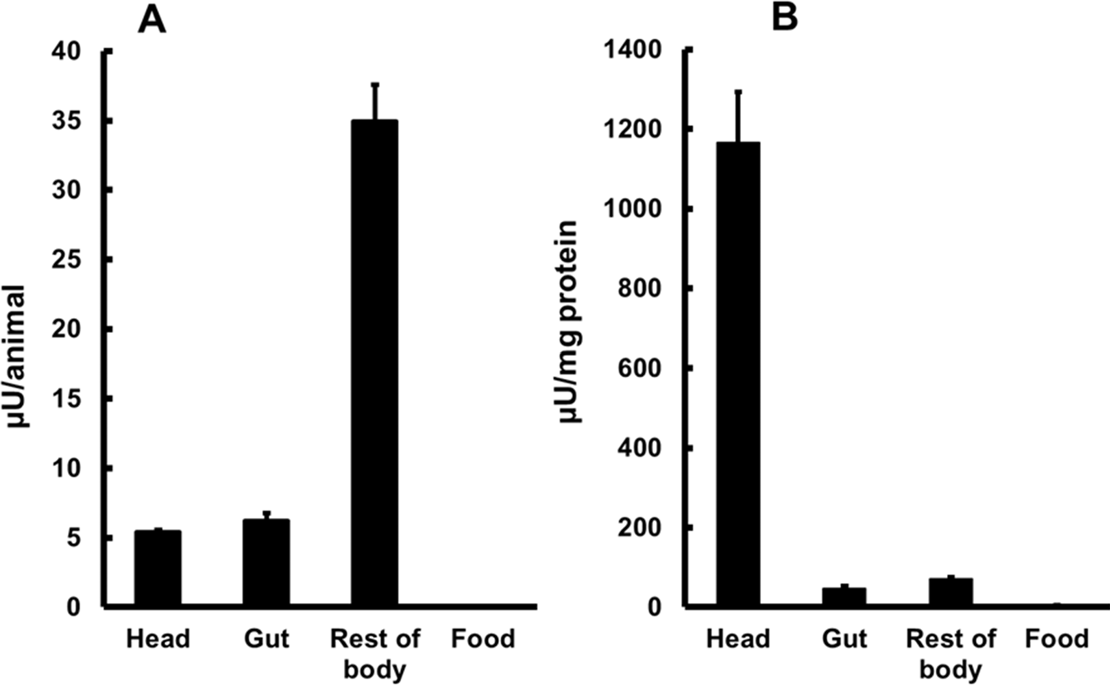
Beta-1,3-glucanase activity in head, gut, rest of body and food of *Aedes aegypti* larvae. (A) Activity per animal (μU/animal). (B) Specific activity (μU/mg protein). Insects were fed with cat food (Whiskas®). Figures are means ± SEM of 2 experiments with 3 samples obtained from 50 insects each.

Since *A*. *aegypti* larvae were able to develop solely on a live *S*. *cerevisiae* diet, we hypothesized that larvae were able to break down the macromolecules from this nutrient source. Because one of the main constituents of the yeast cell wall is beta-1,3-glucan [32], we decided to investigate if *A*. *aegypti* larvae produced beta-1,3-glucanase. Beta-1,3-glucanase activity was present in all parts of 4^th^ instar larvae, with a prevalence in the rest of body and minor activities in the head and gut (Fig 3A). Surprisingly, specific activity (measured as μU/mg protein) in the head was ten times higher than in gut or rest of body (Fig 3B). Activity present in the suspension from containers used to raise the larvae was negligible (Fig 3A), suggesting that activity present in the gut is secreted at this organ and not acquired from food.

**Fig 4:**
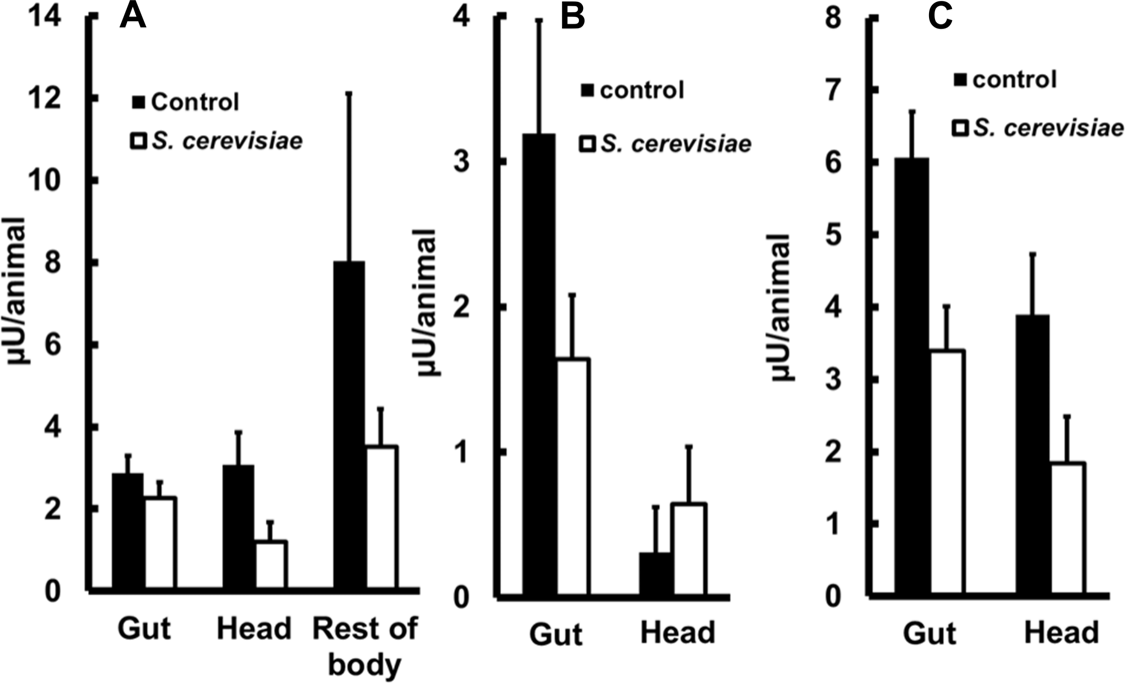
Beta-1,3-glucanase activity in heads, guts and rest of bodies of *Aedes aegypti* larvae fed with different diets. (A) Activities per insect (μU/animal) from soluble fractions of heads, guts and rest of bodies, (B) Activities per insect (μU/animal) from insoluble fractions of heads and guts and (C) Sum of activities in soluble and insoluble fractions of heads and guts. Larvae were fed since hatching exclusively with cat food (Whiskas®) or *Saccharomyces cerevisiae*. live cells. Figures are means ± SEM of 4 experiments with samples obtained from 50 insects each.

After finding significant beta-1,3-glucanase activities in all parts of *A*. *aegypti* 4^th^ instar larvae, we verified whether these activities could be modified (elicited or inhibited) by a diet with live *S*. *cerevisiae* cells. Rearing of *A*. *aegypti* exclusively on live *S*. *cerevisiae* did not result in any significant changes in beta-1,3-glucanase levels in the soluble fraction of all samples tested when compared to controls fed with cat food (p > 0.05, unpaired t-test, n = 4, Fig 4A). We also measured the activity associated with the insoluble fraction of samples, which in the case of gut putatively contains undigested *S*. *cerevisiae* cells and cell walls. The activities in the insoluble fraction of guts and heads were also not changed (p > 0.05, unpaired t-test, n = 4, Fig 4B), as well as the total activity at each tissue (p > 0.05, unpaired t-test, n = 4, soluble + insoluble fractions; Fig 4C).

**Fig 5:**
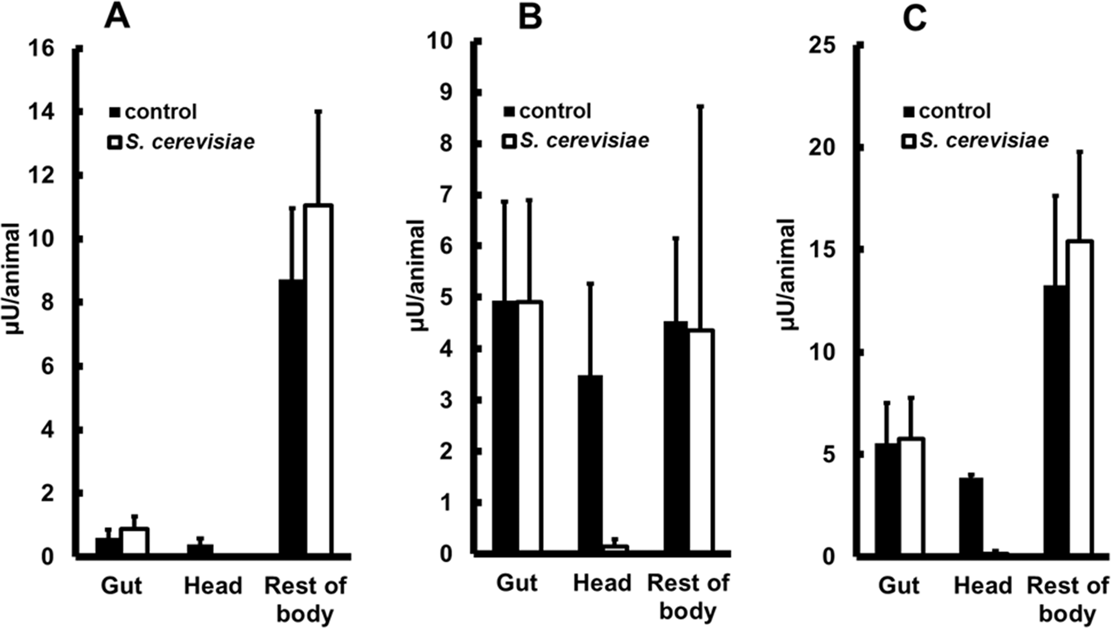
Beta-1,3-glucanase activity in heads, guts and rest of bodies of *Aedes aegypti* larvae fed on different diets. (A) Activities per insect (μU/animal) from soluble fractions of heads, guts and rest of bodies, (B) Activities per insect (μU/animal) from insoluble fractions of heads, guts and rest of bodies, (C) Total activities in heads, guts and rest of bodies (soluble plus insoluble fractions). Larvae were fed since egg hacthing exclusively with cat food (Whiskas®) or autoclaved *Saccharomyces cerevisiae* cells. Figures are means ± SEM of 4 experiments with samples obtained from 50 insects each.

Surface exposure of structural components of the cell wall could be an important factor in possible changes in beta-1,3-glucanase activity in larvae during development when feeding on yeast cells. Nevertheless, activities from insects fed on autoclaved *S*. *cerevisiae* did not differ from controls in all tissues, neither in the soluble fraction (*p* > 0.05, unpaired t-test, n = 4, Fig 5A), the insoluble fraction (*p* > 0.05, unpaired t-test, n = 4, Fig 5B) or in total amount (*p* > 0.05, unpaired t-test, n = 4, Fig 5C). The only remarkable exception on this pattern was the activity in the head, which was significantly lower in larvae fed on autoclaved yeasts compared with controls fed on cat food. This was observed in total as well as both soluble and insoluble fractions (*p* < 0.05, unpaired t-test, n = 4, Fig 5A–5C).

**Fig 6:**
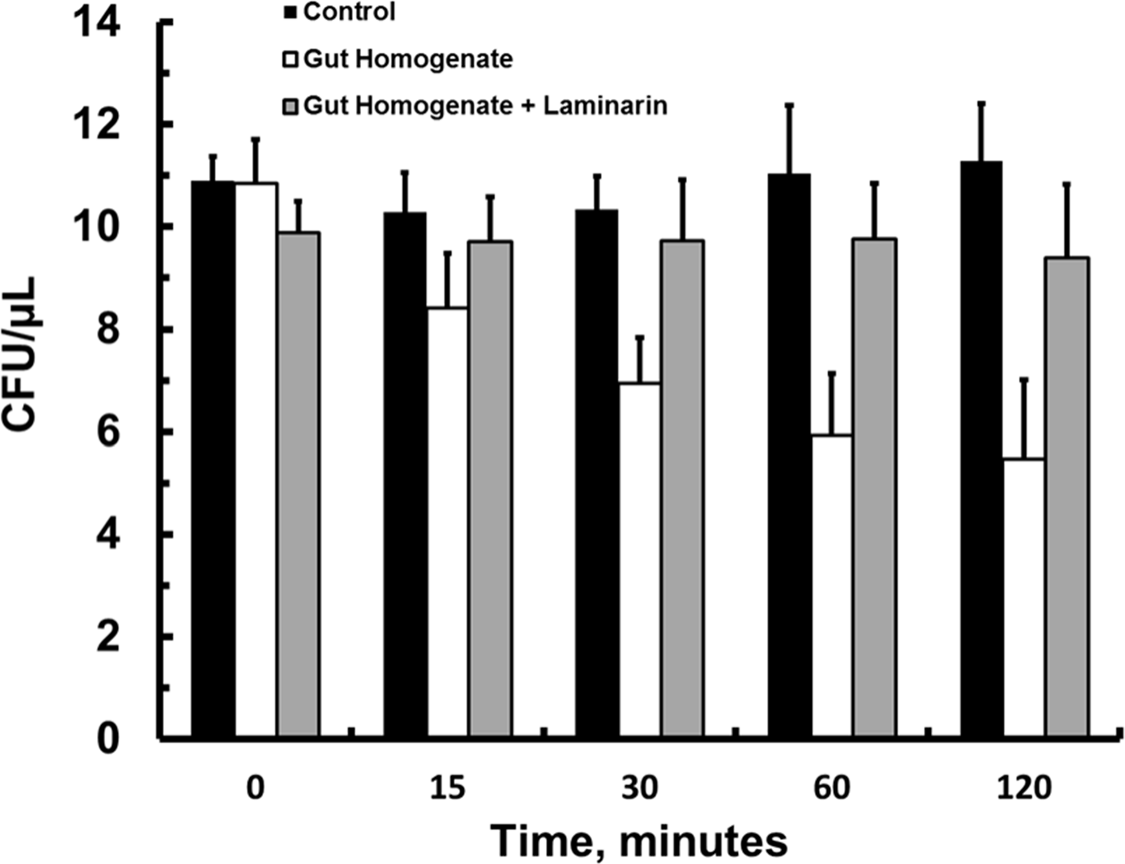
Lysis of *Saccharomyces cerevisiae* live cells in hypotonic media during incubation with soluble fraction of *Aedes aegypti* larvae guts. The cells were incubated in 10 mM citrate-sodium phosphate buffer pH 7. Controls—*Saccharomyces cerevisiae* cells (10 CFUs/uL) in buffer; Gut Homogenates—*S*. *cerevisiae* cells (10 CFUs/uL) incubated with the soluble fraction of *A*. *aegypti* larval midguts (0.1 animal/uL) Gut Homogenates + Laminarin—*S*. *cerevisiae* cells (10 CFUs/uL) incubated with the soluble fraction of *A*. *aegypti* larval midguts (0.1 animal/uL) plus laminarin (1.7%, w/v). Figures are means ± SEM of 3 experiments with 3 samples obtained from 10 insects each.

The presence of a constitutive beta-1,3-glucanase activity in the gut of *A*. *aegypti* larvae raised the question about the real importance of this enzyme in the breakdown of ingested yeast cell walls. These cells were stable under assay conditions (see controls, Fig 6), and incubation of these cells with gut soluble fraction from *A*. *aegypti* larvae resulted in rapid loss of viability (*p* < 0.05, unpaired t-test *vs* controls, n = 9, Fig 6). Addition of laminarin, a beta-1,3-glucan from *Laminaria digitata* and a commercial substrate for beta-1,3-glucanases, to the assay mixture prevented the effect of *A*. *aegypti* larval gut soluble fraction on live *S*. *cerevisiae*. cells (*p* > 0.05, unpaired t-test *vs* controls, n = 9, Fig 6).

## Discussion

Mosquito larvae feed on particulate material, which can include plant debris, algae, protists, and fungal and bacterial cells. In fact, several works supported by evidence derived with microscopes showed the active ingestion of microorganisms by mosquito larvae [16–21]. Sometimes the identification of intact cells inside the larval gut is difficult due to their quick disruption, which seems to be the case for protists. Considering the observed speed of the effect of *A*. *aegypti* larval gut homogenates on *S*. *cerevisiae* cells (20% viability loss in 15 minutes), this could partially explain the poor record of yeast cells inside the gut of culicid larvae.

Culicidae larvae present different modes of feeding. Although classified as filter feeders, sometimes it is hard to distinguish passive ingestion from active selection of food components. In spite of that, yeast cells have already been described as part of the mosquito diet, but there is no evidence about the nutritional importance of these microorganisms in wild larvae. In fact, mosquito larvae seem to be strongly generalist, coping with extreme variations of microbial composition in nursing sites [9]. The data presented in this work showed that *A*. *aegypti* larvae was able to ingest yeast cells, but more experiments should be performed to assess the preference of larvae for *S*. *cerevisiae* over other dietary microbes.

Since *S*. *cerevisiae* sp. live cells were the only source of carbon and nitrogen for *A*. *aegypti* larvae in our experiments, it was expected that they produced enzymes capable of digesting the main polyssaccharides and proteins of this yeast. Digestive chitinases and trypsin-like proteases were already described in mosquito larvae [14, 33–34], but digestion of beta-1,3-glucan, the major yeast cell wall polyssaccharide [32], was never studied in Culicidae. Beta-1,3-glucanase activities were described in cockroaches, termites, grasshoppers, beetles, moth larvae and, recently, in sandfly larvae [33–41]. These are digestive enzymes involved in the breakdown of plant hemicellulose or fungal cell wall disruption. Some insect gut beta-1,3-glucanases have high lytic capacity against yeast cells, being in those cases, endo-beta-1,3-glucanases (E.C.3.2.1.39) [37].

Insects beta-1,3-glucanases are proteins belonging to glycoside hydrolase family 16 [36, 38]. In some insects as termites and moths, GHF16 proteins were incriminated in pathogen recogniton [42–43]. This dual physiological role is evident in the distribution of beta-1,3-glucanase activity in *A*. *aegypti* larvae. Gut activities seem to be constitutive, as it would be expected for a digestive enzyme in holometabolan larvae [44–45]. Head beta-1,3-glucanase seems to be involved in sensing of microbes in ingested food, as autoclaved food resulted in ablation of this enzyme. A similar pattern of expression was observed for lysozymes in *Drosophila* larvae [46]. Activity in the rest of body is putatively involved in defense against pathogens, since digestion does not occur in these tissues and beta-1,3-glucans were never described as intermediate metabolites in animals. In this respect, *A*. *aegypti* larval beta-1,3-glucanases could be homologous to the beta-1,3-glucanases already described in other insects as beetles (gut, [37]) and moths (rest of body, [43]).

Notably, this is the first description of beta-1,3-glucanases in larvae of Culicidae. Beta-1,3-glucanase activity in sand fly *Lutzomyia longipalpis* larvae is putatively related with the active ingestion of fungal cells by this insect [40–41]. The presence of beta-1,3-glucanases in guts of *A*. *aegypti* larvae suggests that fungal and plant hemicelluloses could be regular components in their diet, as this enzyme has these structures as substrates in other insects. In this respect, *A*. *aegypti* larval gut beta-1,3-glucanase could be complementing the chitinase activity already described [33], which is putatively involved in digestion of fungi and other chitin-containing particles.

It is possible that chitinase and beta-1,3-glucanase have complementary roles in fungal cell disruption by mosquito larvae, but the observation that the presence of laminarin (commercial beta-1,3-glucan) in excess prevented lysis of live *S*. *cerevisiae* cells by gut homogenates suggest that beta-1,3-glucanase is essential for disruption of yeast cell walls. This evidence coincides with the predominance of beta-1,3-glucans in fungal cell walls and their structural role [32, 47]. In this respect, beta-1,3-glucanase might be an important enzyme for larval nutrition in *A*. *aegypti* larvae and an interesting target for inhibition, as mammals lack this enzyme (CAZY, www.cazy.org). Additionaly, beta-1,3-glucanase might be an essential enzyme for mosquito larvae feeding on fungi, as mechanical disruption of cells in insect digestion is negligible, and chemical break down of cell wall polysaccharides in necessary to permit access to intracellular nutrient sources as proteins, glycogen and nucleic acids [44–45].

Nevertheless, further molecular characterization of beta-1,3-glucanase activity in *A*. *aegypti* is required, because some insect beta-glycosidases also have activity against laminarin [48] and lytic activity against yeast cells was also reported for glycosidases [49]. However, it is unlikely that beta-glycosidase is the main responsible for lysis in *A*. *aegypti* larvae, because insect glycosidases have low binding affinity for laminarin, and in this case this substrate would constitute a poor competitor in the lytic assay.

Results shown here demonstrate that *A*. *aegypti* can complete development on a diet exclusively of *S*. *cerevisiae* cells. In this respect, these cells must contain all macro and micronutrients which are necessary for mosquito development. This is expected to a certain extent, as yeast extract (*S*. *cerevisiae*) had already been used as an exclusive food source to fully develop *A*. *aegypti* [49]. It is interesting to notice that using *S*. *cerevisiae* live cells we obtained a similar delay in pupation when compared to controls (2 days), but higher percentages of adult emergence (80%) when compared to insects developed on yeast extract (58%) [50]. Our observation that the yeast-based diet has much higher protein and sugar contents than the regular cat food points to a possible deficiency in some essential micronutrient, but more studies are necessary to elucidate this issue.

In a very recent report, it was shown that *Culex pipiens* larvae are able to nourish from yeast cells of several species, including *S*. *cerevisiae* [51]. This fact suggests that the nutritional relation between mosquito larvae and yeasts is at least partly shared among Culicidae. It is likely that the lytic mechanism in *C*. *pipiens* involves the action of a β-1,3-glucanase as in *Aedes*, but this still needs to be confirmed. Interestingly, our data suggest that *S*. *cerevisiae* might be a potential probiotic for mosquito larvae, besides being a promising component for the development of diets for larvae based on microorganisms. This might result in cheaper, pathogen free, and more reproducible diets for these insects, with an important impact in mass rearing which is necessary for the development of new vector control management strategies. Considering that *Drosophila* may also be reared on yeasts [52], there is an interesting nutritional parallel throughout the order Diptera. Diets containing yeast cells may be a starting point to novel strategies for intervention in the metabolism or genetics of mosquitoes. These new approaches might include knockout or mutant yeasts, yeast producing recombinant proteins, GFP tagged peptides or dsRNA.

## Conclusions

*Aedes aegypti* larvae were able to ingest and break down live yeast cells (*S*. *cerevisiae*). Beta-1,3-glucanase activities were present in the head, gut and rest of body of these insects, being involved in yeast digestion (gut) and possible recognition of invading microorganisms (head and rest of body). Beta-1,3-glucanase in the gut and rest of body were not affected by yeast diets, but head activity is suppressed in insects fed on autoclaved cells, suggesting a role in sensing of food-borne microbes. *A*. *aegypti* larval gut beta-1,3-glucanase was essential for lysis of yeast cells, and might be a crucial enzyme when these insects feed solely on this nutrient source.

## Supporting Information

S1 File The file S1_File.xls includes raw data used for the calculations of biological parameters, enzyme activities, sugar and protein quantitatities and yeast cell counts, used in Figs 1–6 and Tables 1 and 2. (XLSX)

## References

[1] Nathan MB, Dayal-Drager R. Recent epidemiological trends, the global strategy and public health advances in dengue WHO, Scientific Working Group–Report on Dengue. Geneva, Switzerland: WHO, 2007; 30–34.

[2] Hayes EB. Zika virus outside Africa. Emerg Infect Dis. 2009, 15:1347–50. 10.3201/eid1509.090442 19788800 PMC2819875

[3] Weaver SC, Reisen WK. Present and future arboviral threats. Antiviral Research. 2010; 85: 328–345. 10.1016/j.antiviral.2009.10.008 19857523 PMC2815176

[4] Paz S. Climate change impacts on West Nile virus transmission in a global context. Philos Trans R Soc Lond B Biol Sci. 2015, 370 (1665). 10.1098/rstb.2013.0561 PMC4342965 25688020

[5] Luz PM, Codeco CT, Medlock J, Struchiner CJ, Valle D, Galvani AP. 2009 Impact of insecticide interventions on the abundance and resistance profile of Aedes aegypti. Epidemiology and Infection 137: 1203–1215. 10.1017/S0950268808001799 19134235

[6] Carvalho DO, Costa-da-Silva AL, Lees RS, Capurro ML. 2014 Two step male release strategy using transgenic mosquito lines to control transmission of vector-borne diseases. Acta Tropica 132: S170–S177 10.1016/j.actatropica.2013.09.023 24513036

[7] Maciel-de-Freitas R, Aguiar R, Bruno RV, Guimaraes MC, Lourenco-de-Oliveira R, Sorgine MHF, Struchiner CJ, Valle D, O'Neill SL, Moreira LA. 2012 Why do we need alternative tools to control mosquito-borne diseases in Latin America? Mem. Inst. Oswaldo Cruz 107: 828–829 22990977 10.1590/s0074-02762012000600021

[8] Villegas LM, Pimenta PFP. 2014 Metagenomics, paratransgenesis and the Anopheles microbiome: a portrait of the geographical distribution of the anopheline microbiota based on a meta-analysis of reported taxa. Mem. Inst. Oswaldo Cruz 109: 672–684. 25185007 10.1590/0074-0276140194 PMC4156461

[9] Clements N. 2000 The Biology of Mosquitoes 3 vol. Wallingford: CABI Publishers.

[10] Chen XG, Mathur G, James AA. Gene expression studies in mosquitoes. Adv Genet. 2008; 64:19–50. 10.1016/S0065-2660(08)00802-X 19161831 PMC2798853

[11] Borovsky D. Biosynthesis and control of mosquito gut proteases. IUBMB Life. 2003, 55:435–41. 14609198 10.1080/15216540310001597721

[12] Douglas AE. The molecular basis of bacterial-insect symbiosis. J Mol Biol. 2014, 426:3830–7. 10.1016/j.jmb.2014.04.005 24735869 PMC4385585

[13] Oviedo MN, VanEkeris L, Corena-Mcleod MDP, Linser PJ. A Microarray-based analysis of transcriptional compartmentalization in the alimentary canal of Anopheles gambiae (Diptera: Culicidae) larvae. Insect Mol. Biol. 2008, 17: 61–72. 10.1111/j.1365-2583.2008.00779.x 18237285

[14] Venancio TM, Cristofoletti PT, Ferreira C, Verjovski-Almeida S, Terra WR. The Aedes aegypti larval transcriptome: a comparative perspective with emphasis on trypsins and the domain structure of peritrophins. Insect Mol Biol. 2009, 18:33–44. 10.1111/j.1365-2583.2008.00845.x 19054160

[15] Kay B. Dengue vector surveillance and control. Current Opinion in Infectious Diseases 1999, 12: 425–432 10.1097/00001432-199910000-00003 17035807

[16] Walker ED, Olds EJ, Merritt RW. Gut content analysis of mosquito larvae (Diptera: Culicidae) using DAPI stain and epifluorescence microscopy. J. Med. Entomol. 1988, 25: 551–554. 2905011 10.1093/jmedent/25.6.551

[17] Merritt RW, Olds EJ, Walker ED. Natural food and feeding behavior of Coquillettidia perturbans larvae. J. Am. Mosq. Control Assoc. 1990, 6: 35–42. 2324723

[18] Ho BC, Khoo HG, Chew LM, Wong KP, Ewert A. Food ingestion and digestive enzymes in larval *Aedes aegypti* and *Ae*. *albopictus* (Diptera: Culicidae). J. Med. Entomol. 1992, 29: 960–964. 1460635 10.1093/jmedent/29.6.960

[19] Khawaled K, Cohen T, Zaritsky A. Digestion of *Bacillus thuringiensis* var. israelensis spores by larvae of *Aedes aegypti*. J. Invertebr. Pathol. 1992, 59: 186–189. 1607667 10.1016/0022-2011(92)90031-x

[20] Avissar YJ, Margalit J, Spielman A. (1994). Incorporation of body components of diverse microorganisms by larval mosquitoes. Journal of the American Mosquito Control Association, 10(1), 45–50. 7912260

[21] Muniaraj M, Arunachalam N, Paramasivan R, Mariappan T, Philip Samuel P, Rajamannar V. Bdelloid rotifer, *Philodina* species in the breeding containers of *Aedes aegypti* and *Aedes albopictus*. Trop. biomed. 2012, 29: 646–649. 23202612

[22] Dillon RJ, Dillon VM. The gut bacteria of insects: Nonpathogenic interactions. Ann. Rev. Entomol. 2004, 49: 71–92 14651457 10.1146/annurev.ento.49.061802.123416

[23] Coon KL, Vogel KJ, Brown MR, Strand MR. 2014 Mosquitoes rely on their gut microbiota for development. Molecular Ecology 23: 2727–2739. 10.1111/mec.12771 24766707 PMC4083365

[24] Joy TK, Arik AJ, Corby-Harris V, Johnson AA, Riehle MA. The impact of larval and adult dietary restriction on lifespan, reproduction and growth in the mosquito *Aedes aegypti*. Experimental gerontology 2010, 45: 685–690. 10.1016/j.exger.2010.04.009 20451597 PMC4181608

[25] Takken W, Smallegange RC, Vigneau AJ, Johnston V, Brown M, Mordue-Luntz AJ, et al Larval nutrition differentially affects adult fitness and Plasmodium development in the malaria vectors Anopheles gambiae and Anopheles stephensi. Parasit Vectors 2013, 6: 345 10.1186/1756-3305-6-345 24326030 PMC4029273

[26] Yahouédo GA, Djogbénou L, Saïzonou J, Assogba BS, Makoutodé M, Gilles JR, et al Effect of three larval diets on larval development and male sexual performance of *Anopheles gambiae* ss. Acta tropica 2014, 132: S96–S101. 10.1016/j.actatropica.2013.11.014 24291460

[27] Sandven P, Lassen J. Importance of selective media for recovery of yeasts from clinical specimens. J Clin. Microbiol. 1999, 37: 3731–2. 10523586 10.1128/jcm.37.11.3731-3732.1999 PMC85742

[28] Lucena SA, Moraes CS, Costa SG, de Souza W, Azambuja P, Garcia ES, Genta FA. Miniaturization of hydrolase assays in thermocyclers. Anal Biochem. 2013, 434: 39–43. 10.1016/j.ab.2012.10.032 23123426

[29] Bradford MM. A rapid and sensitive method for the quantitation of microgram quantities of protein utilizing the principle of protein-dye binding. Anal Biochem. 1976, 72: 248–54. 942051 10.1016/0003-2697(76)90527-3

[30] Smith PK, Krohn RI, Hermanson GT, Mallia AK, Gartner FH, Provenzano M, Klenk DC. Measurement of protein using bicinchoninic acid. Anal. Biochem. 1985, 150: 76–85. 3843705 10.1016/0003-2697(85)90442-7

[31] Dubois M, Gilles KA, Hamilton JK, Rebers P, Smith F. (1956). Colorimetric method for determination of sugars and related substances. Analytical chemistry, 28(3), 350–356.

[32] Orlean P. 2012 Architecture and Biosynthesis of the *Saccharomyces cerevisiae* Cell Wall. Genetics 192, 775–818. 10.1534/genetics.112.144485 23135325 PMC3522159

[33] Souza-Neto JA, Gusmão DS, Lemos FJ. Chitinolytic activities in the gut of *Aedes aegypti* (Diptera:Culicidae) larvae and their role in digestion of chitin-rich structures. Comp Biochem Physiol A Mol Integr Physiol., 2003 136: 717–24. 14613799 10.1016/s1095-6433(03)00224-1

[34] Soares TS, Watanabe RM, Lemos FJ, Tanaka AS. Molecular characterization of genes encoding trypsin-like enzymes from *Aedes aegypti* larvae and identification of digestive enzymes. Gene 2011, 489: 70–5. 10.1016/j.gene.2011.08.018 21914468

[35] Genta FA, Terra WR, Ferreira C. Action pattern, specificity, lytic activities, and physiological role of five digestive beta-glucanases isolated from Periplaneta americana. Insect Biochem Mol Biol. 2003, 33:1085–97. 14563360 10.1016/s0965-1748(03)00121-8

[36] Genta FA, Dumont AF, Marana SR, Terra WR, Ferreira C. The interplay of processivity, substrate inhibition and a secondary substrate binding site of an insect exo-beta-1,3-glucanase. Biochim Biophys Acta. 2007, 1774:1079–91. 17720633 10.1016/j.bbapap.2007.07.006

[37] Genta FA, Bragatto I, Terra WR, Ferreira C. Purification, characterization and sequencing of the major beta-1,3-glucanase from the midgut of *Tenebrio molitor* larvae. Insect Biochem Mol Biol. 2009, 39: 861–74. 10.1016/j.ibmb.2009.10.003 19840850

[38] Lucena SA, Lima LS, Cordeiro LS Jr, Sant'anna C, Constantino R, Azambuja P, et al High throughput screening of hydrolytic enzymes from termites using a natural substrate derived from sugarcane bagasse. Biotechnol Biofuels. 2011, 4:51. 22081987 10.1186/1754-6834-4-51 PMC3245446

[39] Bragatto I, Genta FA, Ribeiro AF, Terra WR, Ferreira C. Characterization of a β-1,3-glucanase active in the alkaline midgut of *Spodoptera frugiperda* larvae and its relation to β-glucan-binding proteins. Insect Biochem Mol Biol. 2010, 40: 861–72. 10.1016/j.ibmb.2010.08.006 20816775

[40] Moraes CS, Lucena SA, Moreira BH, Brazil RP, Gontijo NF, Genta FA. Relationship between digestive enzymes and food habit of *Lutzomyia longipalpis* (Diptera: Psychodidae) larvae: Characterization of carbohydrases and digestion of microorganisms. J Insect Physiol. 2012, 58: 1136–45. 10.1016/j.jinsphys.2012.05.015 22684112

[41] Moraes Cda S, Diaz-Albiter HM, Faria Mdo V, Sant'Anna MR, Dillon RJ, Genta FA. 2014 Expression pattern of glycoside hydrolase genes in Lutzomyia longipalpis reveals key enzymes involved in larval digestion. Front Physiol. 5:276 10.3389/fphys.2014.00276 25140153 PMC4122206

[42] Bulmer MS, Bachelet I, Raman R, Rosengaus RB, Sasisekharan R. 2009 Targeting an antimicrobial effector function in insect immunity as a pest control strategy. Proc Natl Acad Sci U S A. 106:12652–7. 10.1073/pnas.0904063106 19506247 PMC2722268

[43] Pauchet Y, Freitak D, Heidel-Fischer HM, Heckel DG, Vogel H. 2010 Glucanase activity in a glucan-binding protein family from Lepidoptera. Journal of Biological Chemistry 284, 2214–2224. 10.1074/jbc.M806204200 19033442

[44] Terra WR, Ferreira C. Insect digestive enzymes: properties, compartmentalization and function. Comp. Biochem. Phys. 1994, 109B, 1–62.

[45] Terra WR, Ferreira C. Biochemistry of digestion In: Gilbert L.I., Iatrov K., Gill S.S. (Eds.), Comprehensive Molecular Insect Science. Biochemistry and Molecular Biology 2005, vol. 4 Elsevier, Oxford, pp. 171–224.

[46] Daffre S, Kylsten P, Samakovlis C, Hultmark D. The lysozyme locus in *Drosophila melanogaster*: an expanded gene family adapted for expression in the digestive tract. Mol Gen Genet. 1994, 242(2): 152–162. 8159165 10.1007/BF00391008

[47] Ruiz-Herrera J. 1991 Biosynthesis of beta-glucans in fungi. Antonie Van Leeuwenhoek. 60: 72–81. 1839492 10.1007/BF00572695

[48] Ferreira AH, Marana SR, Terra WR, Ferreira C. 2001 Purification, molecular cloning, and properties of a beta-glycosidase isolated from midgut lumen of Tenebrio molitor (Coleoptera) larvae. Insect Biochem Mol Biol. 31: 1065–76. 11520685 10.1016/s0965-1748(01)00054-6

[49] Rombouts FM, Phaff HJ. 1976 Lysis of yeast cell walls. Lytic beta-(1–3)-glucanases from Bacillus circulans WL-12. Eur J Biochem. 63: 121–30. 4310 10.1111/j.1432-1033.1976.tb10214.x

[50] Timmermann SE, Briegel H. 1996 Effect of plant, fungal and animal diets on mosquito development. Entomologia Experimentalis et Applicata 80:173–176.

[51] Steyn A, Roets F, Botha A. 2016 Yeasts Associated with *Culex pipiens* and *Culex theileri* Mosquito Larvae and the Effect of Selected Yeast Strains on the Ontogeny of *Culex pipiens*. Microbial Ecology (in press). 10.1007/s00248-015-0709-1 26573833

[52] Ashburner M. Drosophila A laboratory handbook. Cold Spring Harbor Laboratory Press, 1989

